# The effect of resistance training programs on lean body mass in postmenopausal and elderly women: a meta-analysis of observational studies

**DOI:** 10.1007/s40520-021-01853-8

**Published:** 2021-04-20

**Authors:** Ewan Thomas, Ambra Gentile, Nemanja Lakicevic, Tatiana Moro, Marianna Bellafiore, Antonio Paoli, Patrik Drid, Antonio Palma, Antonino Bianco

**Affiliations:** 1 Sport and Exercise Sciences Research Unit, University of Palermo, Via Giovanni Pascoli 6, 90144 Palermo, Italy; 2 PhD Program in Health Promotion and Cognitive Sciences, University of Palermo, Via Giovanni Pascoli 6, 90144 Palermo, Italy; 3 Department of Biomedical Sciences, University of Padova, Via Marzolo 3, 35031 Padova, Italy; 4 Faculty of Sport and Physical Education, University of Novi Sad, Lovćenska 16, 2110 Novi Sad, Serbia

**Keywords:** Hypertrophy, Resistance training, Woman, Elderly, Post-menopausal

## Abstract

Aging and menopause are associated with morphological and functional changes which may lead to loss of muscle mass and therefore quality of life. Resistance training (RT) is an effective training mode to increase muscle mass. We reviewed the existing literature to identify studies implementing RT protocols and evaluating muscle hypertrophy exclusively in healthy, postmenopausal and elderly women. Participants’ age range was comprised between 50 and 80 years. The primary outcome observed was muscle hypertrophy. Fat mass was also evaluated, if available. PubMed and Web of Science were the screened database, and original articles written in English and published from 2000 up to 2020 were included. 26 articles were considered eligible and included. Quality assessment revealed a “moderate quality” of the included studies, however the majority of studies was able to reach level 4 of evidence and on overall grade of recommendation C. In total, data from 745 female participants subjected to different forms of resistance training were considered. Heterogeneity across studies was present regarding study design, intervention length (mean 16 weeks), training frequency (3 d/w), no. of exercises (*n* = 7.4) and participants’ age (65.8 ± 4.9 years). Small-to-moderate significant increases (*k* = 43; SMD = 0.44; 95% CI 0.28; 0.60; *p* < 0.0001) of lean body mass were observed in post-menopausal and elderly women, regardless of age, intervention period, weekly training frequency and no. of exercises. No effects were noted for fat mass (*k* = 17; SMD = 0.27; 95% CI − 0.02; 0.55; *p* = 0.07). Studies need to concentrate on providing information regarding training parameters to more effectively counteract the effects of aging and menopause on skeletal muscle mass.

**Supplementary Information:**

The online version contains supplementary material available at 10.1007/s40520-021-01853-8.

## Introduction

Muscle hypertrophy is defined as an increase in the cross-sectional area of a muscle due to the increase in muscle protein synthesis and contractile tissue [1, 2]. Muscle hypertrophy is a multifaceted phenomenon, that is founded on mechanical stimulation, as well as metabolic and endocrine processes that have been shown to impact gene transcription via different signaling pathways [2]. Mechanical loading, in particular, leads to a number of intracellular actions that ultimately regulate gene expression and protein synthesis via mTORC1 pathway activation [3–5]. Therefore, an effective strategy to promote muscle hypertrophy is represented by long-term resistance training (RT) in both men and women of different ages [6–8]. Variables such as exercise intensity, exercise frequency, rest periods and training volume can be manipulated in order to maximize the magnitude of the effect on muscle hypertrophy and strength [3]. Recent studies have shown that muscle hypertrophy is also associated with strength gains not only in young and middle aged adults, but also in older men and women [9]. This is of particular importance since declines in muscle mass and strength are observed due to aging [10, 11]. The age-related loss of muscle mass and strength can lead to physical disability and frailty [12, 13] and overall is associated with an increased risk of falls [14–16]. Multiple studies have found a link between low levels of muscle mass and low functional capacity [17, 18]. In addition, since muscle is a very metabolically active tissue, metabolic disorders associated with aging, such as diabetes, osteoporosis or decrease in testosterone and growth hormone levels may frequently occur [19, 20]. Therefore, muscle mass loss represents a significant problem for older adults.

Muscle mass loss is also associated to menopause, since a physiological hormonal change is present due to menstruation cessation [21]. In particular, a decline in estrogen concentration has detrimental effects on skeletal muscle mass and functionality, leading to reduced bone mass density, redistribution of fat to the visceral area and increased risk of cardiovascular events [21, 22]. Notably, post-menopausal women with reduced skeletal muscle mass have a 2.1 higher risk of falling and a 2.7 times greater risk of sustaining a fracture compared to women with preserved muscle mass [23].

RT can be used as a potential method of offsetting decline in muscle mass and strength, as improvements in muscle mass have been detected in postmenopausal, middle-aged and older women after RT [24–28].

To our knowledge, despite abundant evidence with regards to variance in response to RT in men and women, there are not enough original investigations able to provide specific guidelines for post-menopausal and elderly women in order to optimize maximal muscle gains. Thus, the main objective of this study is to review the existing literature to identify and analyze current evidence with regards to RT protocols aiming to induce muscle hypertrophy in the post-menopausal and elderly population.

## Methods

The manuscript followed the Preferred Reporting Items for Systematic Reviews and Meta-Analyses (PRISMA) statement [29].

### Search strategy and study selection

The databases PubMed (NLM), Web of Science (TS) and Scopus were used to perform a comprehensive search for relevant articles published between January 1, 2000 and November 11, 2020. The search strategy included terms in the search field “title” and/or “topic” and “abstract” of each database. The final searches were then executed using the appropriate specifications of each database using the PICOS format (See supplementary file).

### Eligibility criteria

To be included studies: (1) had to include healthy women aged between 50 and 80 years of age, (2) with no physical, mental or neurological disorders, (3) interventions based solely on RT programs conducted in postmenopausal and older adult women, (4) pre and post-intervention results and (5) published in English. Publications were excluded if: (1) Reviews, meta-analysis, abstracts, scientific conference works, posters, citations, letters to the editor, books, statements, (2) non-peer reviewed journal articles, (3) commentaries, (4) together with studies reported in languages other than English.

The primary outcome was identified in a change in muscle mass, i.e. hypertrophy measured by dual-energy X-ray absorptiometry (DXA), magnetic resonance imaging (MRI), ultrasound imaging (USI), bioelectric impedance analysis (BIA) or other valid method able to detect changes in lean tissue.

### Study record

**Fig. 1:**
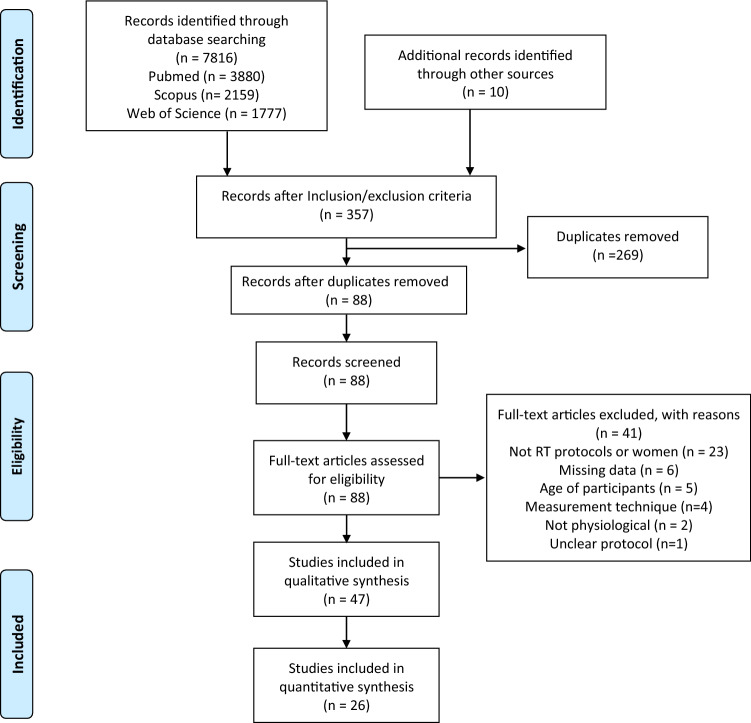
PRISMA flow diagram describing the inclusion process of the retrieved articles

Search results were uploaded to EndNote X 8.1 (Clarivate Analytics, Jersey, UK) and duplicates were removed. Two independent investigators (ET and AG) screened the titles and abstracts for relevance based on inclusion criteria for this systematic review. Full text of articles were also screened if title and abstract were not sufficient to determine eligibility. Disagreement of article inclusion was resolved by discussion and consensus with a third investigator (AB). The screening process has been summarized in a PRISMA flow diagram (Fig. 1). Three tables were created to extract relevant study data using a Microsoft Excel (Microsoft Corp, Redmond, Washington) spreadsheet. In the first table, information on the first author and year of publication, sample size, mean age and standard deviation, exercise intensity, duration of the intervention, and exercise frequency per week were shown. The second table consisted of test battery used, pre-intervention values, post-intervention values and potential discrepancy (possible incremental change) regarding lean body mass. The third table included data relevant to fat mass, when available. Authors were contacted via email if important data was missing from a particular study. If the contacted author did not respond to the questions asked about the specifics of a study, these were excluded from the review. The WebPlotDigitizer (version 4.2) software, was used to extrapolate information from figures, if relevant information for this review was not included in tables or the main text of the manuscripts.

### Risk of bias assessment

For risk of bias assessment, we used The Downs and Black checklist [30] which assesses the quality of original research articles in order to synthesize evidence from quantitative studies for public health purposes. This checklist contains 27 ‘yes’-or- ‘no’ questions across five domains. It provides both an overall score for study quality and a numeric score out of a possible 32 points. The five domains comprise questions concerning study quality, external validity, study bias, confounding and selection bias, and power of the study.

Two independent researchers completed the Downs and Black checklist (ET and AG) for included articles to determine the quality of each study. The maximum score a study can receive is 32, with higher scores denoting greater quality. The studies were then separated into groups and labeled as ‘high quality’ (score 23–32), ‘moderate quality’ (score 19–22), ‘lower quality’ (score 15–18) or ‘poor quality’ (≤ 14). Interclass correlation statistical method was used to determine inter-rater reliability. Quality of evidence was obtained by the study design and by the Downs and Black score (Supplementary File). Levels of evidence and grades of recommendation have been also included for each study. The guidelines from the Centre for Evidence-Based Medicine (CEBM, http://www.cebm.net, Last accessed 12/02/2021) regarding the grading for evidence and the guidelines of the American Society of Plastic Surgeons (https://www.plasticsurgery.org/documents/medical-professionals/health-policy/evidence-practice/ASPS-Scale-for-Grading-Recommendations.pdf, last accessed 12/02/2021) regarding grading of recommendation were adopted. A supplementary table has been provided with the results of the quality assessment (Supplementary File).

### Data synthesis

The included studies were first synthesized through a narrative description of the features deriving from each study. Afterwards, the essential characteristics of the studies were represented in tables, where means, standard deviations and the percentage difference between pre- and post- condition were reported. Descriptive statistics of the studies was performed through Jamovi (version 1.6.3.0, The jamovi project, 2020).

Concerning the metanalytic synthesis, the considered outcomes were body lean mass and body fat mass. For each study, means and standard deviations were noted, together with the assessment method used to detect the outcomes (BIA, DXA, MRI, US).

Meta-analysis was performed through the package metafor of the R software (version 3.5.3), using the random effect model on the Standardized Mean Difference (SMD) between pre- and post- measurements. The effects were then represented through a forest plot, and to detect potential influences of publication bias, a funnel plot was performed. The heterogeneity of the studies was estimated through the Cochrane’s Q, and a moderator analysis with age (defined by two categories: below 65 and above 65 years of age), intervention length, number of weekly sessions and number of exercises proposed was planned. Finally, to detect the validity of the results included in the meta-analysis, two sensitivity analyses considering measurement tool and study quality were performed. In the first one, only results derived from DXA were included in the meta-analysis, while in the second one, the effects from poor quality studies were considered.

## Results

### Search outcomes

The electronic database search yielded 7816 articles (Pubmed = 3880, Scopus = 2159, Web Of Science = 1777). Ten additional articles were identified from other sources as potentially relevant. A total of 7459 irrelevant articles were excluded based on title and abstract and further 269 duplicate records were removed. Preliminary search results and duplicate removal provided a total of 88 articles. First steps in the initial assessment of articles were to screen in detail the titles and the abstracts to identify only relevant articles. Subsequently, these full-text articles were screened for relevance and during this process, 41 articles were removed since these were not eligible according to the inclusion criteria. Twenty one articles were excluded from the study, mainly due to inadequate exposure during the intervention, meaning that methods other than RT were incorporated in the study, or ineligible outcomes were detected which did not fit the inclusion criteria of the review. Finally, 26 articles met the inclusion criteria and were included in the study [9, 31–55]. Amongst included studies, the average number of participants per study was 23 while the mean duration of the studies was 16 weeks. On average, participants underwent 3 RT sessions per week including 7.5 exercises, at an intensity of ⁓60% of their 1RM, performing between 9 and 16 repetitions per set. Articles differed greatly in terms of study design, intervention length, follow-up period, subjects’ age, observed outcomes and measurement of the main outcomes.

Table 1 provides a summary of the studies included in the review. All the included records were original research articles (*n* = 26). In total, data from 745 participants were pooled for this review. Studies ranged from 8 [31] to 78 participants [54]. The included articles were published over 20 years from 2000 to 2020.

**Table 1:** Table describes the training modalities of the retrieved studies

Study	Participants	Age (years)	Intervention period (wks)	Training module	Training intensity (1RM)	Reps	Training frequency (d/wk)	No. of exercise/ training
Botero (2013) [41]	23	63.0 ± 4.4	54	FBRT	NA	10–12	2	3
Cannon (2007) [31]	8	69.8 ± 6.6	10	LBRT	50–75%	10	3	2
Churchward-Venne (2015) [50]	44	72.6 ± 0.9	24	FBRT	50–75%	8–15	3	6
Coelho-Júnior (2019) [51]	22	66.8 ± 0.4	24	FBRT	NA	8–15	2	9
Correa (2016) [32]	12	64 ± 5	12	FBRT	NA	8–20	2	9
Cunha (2020) [33]	20	68.6 ± 4.4	12	FBRT	NA	10–15	3	8
Cunha (2020) [33]	21	70.1 ± 5.9	12	FBRT	NA	10–15	3	8
de Oliveira Júnior (2020) [42]	19	58.5 ± 8.0	12	LBRT	NA	8–12	3	4
de Oliveira Júnior (2020) [42]	18	59.3 ± 8.4	12	LBRT	NA	8–12	3	4
Dib (2020) [48]	45	69.2 ± 5.5	12	FBRT	NA	5/10/15	3	8
dos Santos (2016) [43]	33	68.7 ± 5.7	12	FBRT	NA	10–15	3	8
Gambassi (2016) [55]	26	65.0 ± 3.0	12	FBRT	NA	8	2	8
Hakkinen (2001) [9]	10	64 ± 3	21	LBRT	40–80%	8–20	2	6–7
Janzen (2006) [49]	26	55.3 ± 7.4	26	FBRT	50–60%	12	3	11
Leenders (2013) [52]	24	71 ± 1	24	FBRT	60–80%	8–15	3	6
Nascimento (2018) [34]	21	67.8 ± 4.6	12	FBRT	NA	10–15	2	8
Nascimento (2018) [34]	24	69.2 ± 5.7	12	FBRT	NA	10–15	3	8
Nunes (2020) [33]	66	68.8 ± 4.6	12	FBRT	NA	10–15	3	8
Orsatti (2008) [35]	27	57.8 ± 8	16	FBRT	40–80%	8–15	3	8
Orsatti (2012) [36]	22	56.7	39	FBRT	60–80%	8–20	2	8
Pina (2019) [44]	23	65.4 ± 4.4	12	FBRT	NA	10–20	2	8
Pina (2019) [44]	24	64.9 ± 4.6	12	FBRT	NA	10–20	3	8
Pina (2020) [45]	18	68 ± 6	24	FBRT	NA	10–15	2	8
Pina (2020) [45]	19	69 ± 7	24	FBRT	NA	10–15	3	8
Rabelo (2011) [54]	78	67.1 ± 5.9	24	FBRT	60–80%	8–12	3	6
Radaelli (2014) [37]	14	64.7 ± 2.1	6	FBRT	NA	15–20	2	10
Radaelli (2014) [37]	13	64.1 ± 1.8	6	FBRT	NA	15–20	2	10
Ribeiro (2017) [38]	25	67.6 ± 5.1	8	FBRT	NA	8–12	3	8
Santos (2017) [39]	23	69.6 ± 6.4	8	FBRT	NA	10–15	3	8
Thiebaud (2013) [40]	14	61 ± 5	8	FBRT	10–90%	10–30	3	7
Tomeleri (2019) [46]	14	69.7 ± 5.7	12	FBRT	~60%	10–15	3	8
Tomeleri (2019) [46]	15	71.4 ± 6.0	12	FBRT	~60%	10–15	3	8
Vieira (2020) [47]	20	64.0 ± 3.5	16	FBRT	NA	6–14	2	8
Tot./Mean ± std.dv	745	65.8 ± 4.9	16	–	~60%	9–16	3	7.4

*LBRT* lower body resistance training, *UPRT* upper body resistance training, *FBRT* full body resistance training, *1RM* one repetition maximum, *NA* not available

In regards to the measured outcomes, of the 26 articles included in the study, eighteen articles used DXA scanners [33, 34, 36, 38–46, 48–50, 52, 54] to determine whether muscle hypertrophy was evident or not after the intervention, four articles used a BIA [35, 51, 53, 55], two articles used MRI [9, 31] scans and two articles used USI [32, 37]. It is important to note that these screening tools were used to measure different regions of the body which had been exposed to the RT.

Of the different body regions exposed to RT, 23 studies implemented full-body RT [32–41, 43–55], and 3 implemented lower body RT [9, 31, 42]. Thus, only the areas of the body which underwent the RT intervention have been examined by the afore mentioned screening tools.

Table 2 provides a summary of the results of the primary outcomes of interest, while Table 3 provides measures of body fat mass when these were available. Pre and post-intervention values have been identified for each study, and differences between the two have been outlined, when possible. As for the testing methods, there was high heterogeneity in terms of reporting interventions. Some studies reported absolute values, others the percentage of lean bone-free muscle tissue while others reported it as a muscle mass index. Alternatively, some studies reported kilograms of muscle mass prior and after the intervention. Therefore, in order to quantify and normalize the effect of each intervention, percentage differences were determined. A mean increase of 4.8% of lean body mass and a mean decrease of 2.1% of fat body mass have been observed across the retrieved studies.

**Table 2:** The table describes testing methods and differences compared to baseline values regarding lean body mass of each study

Study	Measurement tool	Pre-intervention value	Post-intervention value	Δ hypertrophy	% Δ	SMD	1RM change
Botero (2013) [41]	DXA	38.9 ± 0.9 kg	39.5 ± 1.1 kg	0.6 kg	1.5	0.59	BP 9.9 kg
Cannon (2007) [31]	MRI	51 ± 6 cm^2^	57 ± 7 cm^2^	6 cm^2^	12	0.87	NA
Churchward-Venne (2015) [50]	DXA	41.8 ± 0.7 kg	43.0 ± 0.5 kg	1.2 kg	2.9	1.95	LP 31 kg
Coelho-Júnior (2019) NP [51]	BIA	37.7 ± 3.1 kg	40.1 ± 1.6 kg	2.4 kg	6.4	0.94	KE 19.1 kg
Coelho-Júnior (2019) DUP [51]	BIA	37.5 ± 3.8 kg	39.0 ± 4.0 kg	1.5 kg	4.0	0.37	KE -3 kg
Correa (2016) RT [32]	US	686.3 ± 200.1 cm^3^	850.1 ± 185.2 cm^3^	163.8 cm^3^	23.9	0.82	KE 24.3 kg/ BC 4.1 kg
Correa (2016) DT-ReT [32]	US	691.1 ± 172.5 cm^3^	791.1 ± 162.5 cm^3^	100.0 cm^3^	14.5	0.58	KE 15 kg/ BC 2.8 kg
Cunha (2020) SS [33]	DXA	34.1 ± 5.53 kg	36.2 ± 5.69 kg	2.1 kg	6.2	0.37	NA
Cunha (2020) MS [33]	DXA	28.6 ± 4.23 kg	30.6 ± 4.14 kg	1.9 kg	7	0.47	NA
de Oliveira Júnior (2020) [42] LV	DXA	4.6 ± 0.7 kg	4.8 ± 0.8 kg	0.2 kg	4.3	0.26	KE 19.1 N.m
de Oliveira Júnior (2020) [42] HV	DXA	4.4 ± 0.8 kg	4.8 ± 1.0 kg	0.4 kg	9.1	0.43	KE 6.6 N.m
Dib (2020) [48] MJ-SJ	DXA	18.3 ± 1.9 kg	18.5 ± 2.0 kg	0.2 kg	1.1	0.10	CP 2.4 kg / KE 5.3 kg
Dib (2020) [48] SJ-MJ	DXA	17.7 ± 3.2 kg	17.9 ± 3.2 kg	0.2 kg	1.1	0.06	CP 3.6 kg/ KE 5.2 kg
Dib (2020) [48] ALT	DXA	18.1 ± 2.7 kg	18.3 ± 2.7 kg	0.2 kg	1.1	0.07	CP 1.9 kg/ KE 6.6 kg
dos Santos (2016) [43]	DXA	42.6 ± 5.7 kg	42.9 ± 5.7 kg	0.3 kg	0.7	0.05	BP 9.8 kg
Gambassi (2016) [55]	BIA	38.0 ± 1.5 kg	42.0 ± 1.4 kg	4 kg	10.5	2.72	KE 7 kg
Hakkinen (2001) [9]	MRI	52.2 ± 6.95 cm^2^	56.4 ± 8.1 cm^2^	4.2 cm^2^	8	0.53	31 kg
Janzen (2006) BL [49]	DXA	35.2 ± 5.4 kg	36.4 ± 6.1 kg	1.2 kg	3.4	0.22	LP 34 kg
Janzen (2006) UL [49]	DXA	35.3 ± 4.4 kg	36.8 ± 4.9 kg	1.5 kg	4.2	0.31	LP 26 kg
Leenders (2013) [52]	DXA	42.5 ± 0.9 kg	43.7 ± 1.0 kg	1.2 kg	2.8	1.24	NA
Nascimento (2018) [34]2	DXA	20.1 ± 3.5 kg	21.2 ± 4.1 kg	1.1 kg	5.5	0.28	NA
Nascimento (2018) [34] 3	DXA	19.4 ± 3.4 kg	20.6 ± 3.2 kg	1.2 kg	6.2	0.36	NA
Nunes (2020) [33]	BIA	17.1 ± 2.6 kg	17.7 ± 2.7 kg	0.6 kg	3.5	0.22	CP 4.8 kg/ KE 5.5 kg
Orsatti (2008) [35] RT	BIA	18.8 ± 3.3 kg	20.6 ± 3.6 kg	1.8 kg	9.6	0.51	BP 9.5 kg / LP 4.4 kg
Orsatti (2012) [36]< 5%	DXA	31.9 ± 5.5 kg	32.8 ± 5.4 kg	0.1 kg	0.3	0.16	NA
Pina (2019) [44] 2	DXA	35.5 ± 3.5 kg	36.1 ± 3.3 kg	0.6 kg	1.7	0.17	BP 3.4 kg
Pina (2019) [44] 3	DXA	34.7 ± 3.6 kg	35.3 ± 4.1 kg	0.6 kg	2.3	0.15	BP 3.3 kg
Pina (2020) [45] 2	DXA	19.3 ± 3.7 kg	20.1 ± 3.7 kg	0.8 kg	4.1	0.21	CP 6.1 kg/ KE 7.8 kg
Pina (2020) [45] 3	DXA	19.8 ± 3.5 kg	20.1 ± 3.3 kg	0.3 kg	1.5	0.09	CP 7 kg/ KE 9.4 kg
Rabelo (2011) [54]	DXA	36.4 ± 4.0 kg	37.1 ± 4.2 kg	0.7 kg	1.9	0.17	BP 17.4 kg/ KE 25.9 kg
Radaelli (2014) [37] SS VL	US	16.6 ± 4 mm	17.4 ± 4.8 mm	0.8 mm	4.9	0.18	7 kg
Radaelli (2014) [37] SS RF	US	14.9 ± 4.5 mm	15.6 ± 4.2 mm	0.7 mm	4.3	0.16	7 kg
Radaelli (2014) [37] MS VL	US	17.2 ± 3 mm	18.3 ± 3.1 mm	1.1 mm	5.2	0.35	9.5 kg
Radaelli (2014) [37] MS RF	US	15.1 ± 2.8 mm	16.0 ± 2.4 mm	0.9 mm	5.5	0.33	9.5 kg
Ribeiro (2017) [38] TDTR	DXA	16.6 ± 1.7 kg	17.1 ± 1.8 kg	0.5 kg	3	0.28	12.4 kg
Ribeiro (2017) [38] PRTR	DXA	16.6 ± 1.6 kg	16.9 ± 1.7 kg	0.3 kg	1.8	0.18	11.4 kg
dos Santos (2016) [43]	DXA	18.7 ± 2.9 kg	19.2 ± 2.8 kg	0.5 kg	2.5	0.17	1.9 kg
Thiebaud (2013) [40] MH	DXA	40.9 ± 6.4 kg	41.2 ± 6.2 kg	0.3 kg	0.7	0.05	LP 13.5 kg
Thiebaud (2013) [40] LI-BFR	DXA	41.3 ± 4.5 kg	42.2 ± 4.5 kg	0.9 kg	2.2	0.19	LP 7.6 kg
Tomeleri (2019) [46] SJ	DXA	34.2 ± 3.2 kg	35.8 ± 1.8 kg	1.6 kg	4.7	0.21	CP 4.2 kg/ KE 8 kg
Tomeleri (2019) [46] MJ	DXA	33.9 ± 1.1 kg	36.0 ± 1.2 kg	2.1 kg	6.2	1.78	CP 5.4 kg/ KE 10.8 kg
Vieira (2020) [47] HS	DXA	37.84 ± 3.80 kg	38.05 ± 3.57 kg	0.21 kg	0.6	0.06	BP 2.9 kg/ LP 30.4 kg
Vieira (2020) [47]VHS	DXA	34.15 ± 2.11 kg	34.60 ± 2.09 kg	0.45 kg	1.3	0.20	BP 5.3 kg / LP 50.0 kg
Mean	–	–	–	–	4.8	0.44	BP 7.7 kg/ CP 4.4 kg/ KE 10.8 kg/ LP 24.6 kg

*1* *RM* one repetition maximum, *BC* biceps curl, *BIA* bioelectric impedance analysis, *BL* bilateral, *BP* bench press, *CP* chest press, *DUP* ondulating periodized, *DXA* dual-energy Xray absorptiometry, *DT-ReT* detraining-retraining, *HS* high supervised, *HV* high volume, *KE* knee extensors, *LI-BFR* low load blood flow restriction exercise, *LP* leg press, *LV* low volume, *MH* moderate to high intensity elastic band resistance exercise, *MJ* multi joint, *MRI* magnetic resonance imaging, *MS* multiple-set resistance training, *MV* muscle volume, *NA* not available, *NP* non periodized, *PR* pyramid, *RF* rectus femoris, *RF* repetition to failure, *RT* resistance training, *SF* skin folds, *SJ* single joint, *SS* single-set resistance training, *TD* traditional, *UL* unilateral, *US* ultrasound imaging, *VHS* very high supervised, *VL* vastus lateralis*; < 5%* < 5% of truck fat gain

**Table 3:** The table describes testing methods and differences compared to baseline values regarding fat mass of each study

Study	Measurement tool	Pre-intervention value	Post-intervention value	FMΔ	% Δ	SMD
Botero (2013) [41]	DXA	24.27 ± 1.32 kg	23.54 ± 1.37 kg	− 0.73 kg	− 3	0.53
Coelho-Júnior (2019) [51] NP	BIA	41.9 ± 6.3 kg	41.7 ± 3.6 kg	− 0.2	0.5	0.04
Coelho-Júnior (2019) [51]DUP	BIA	39.1 ± 8.5 kg	39.1 ± 8.7 kg		0	0
de Oliveira Júnior (2020) [42] LV	DXA	28.99 ± 9.28 kg	–	–	–	–
de Oliveira Júnior (2020) [42] HV	DXA	28.87 ± 8.78 kg	–	–	–	–
Dib (2020) [48] MJ-SJ	DXA	25.8 ± 8.9 kg	25.8 ± 9.0 kg	0	0	0
Dib (2020) [48] SJ-MJ	DXA	24.5 ± 7.2 kg	24.8 ± 7.5 kg	0.3	1.2	0.04
Dib (2020)[48] ALT	DXA	24.9 ± 8.7 kg	25.0 ± 8.7 kg	0.1	0.4	0.01
dos Santos (2016) [43]	DXA	25.3 ± 9.4 kg	24.9 ± 9.7 kg	− 0.4 kg	− 1.6	0.04
Gambassi (2016) [55]	BIA	23.0 ± 1.2 kg	20.0 ± 1.1 kg	− 3	− 13	2.56
Leenders (2013) [52]	DXA	21.2 ± 0.9 kg	20.3 ± 0.9 kg	− 0.9	− 4.2	0.98
Nunes (2020) [33]	BIA	28.5 ± 12.8 kg	28.4 ± 12.7 kg	− 0.1	− 0.4	0.01
Orsatti (2008) [35] RT	BIA	35.6 ± 8.1%	34.9 ± 8.3%	− 0.7%	− 2	0.08
Orsatti (2012) [36] < 5%	DXA	12.9 ± 3.8 kg	12.8 ± 3.9 kg	− 0.1 kg	− 0.8	0.03
Pina (2019) [44] 2	DXA	25.1 ± 6.4 kg	24.9 ± 6.9 kg	− 0.2 kg	− 0.8	0.03
Pina (2019) [44] 3	DXA	24.3 ± 6.8 kg	23.6 ± 6.7 kg	− 0.7 kg	− 2.9	0.10
dos Santos (2016) [43]	DXA	24.81 ± 9.4 kg	24.17 ± 9.2 kg	− 0.64 kg	− 2.6	0.07
Vieira (2020) [47] HS	DXA	30.21 ± 9.31 kg	30.21 ± 10.03 kg	0	0	0
Vieira (2020) [47]VHS	DXA	26.94 ± 6.72 kg	25.43 ± 6.64 kg	− 1.51	− 5.6	0.22
Mean	–	–	–	–	− 2.1	0.27

*ALT* alternating upper and lower body, *BIA* bioelectric impedance analysis, *DUP* periodized, *DXA* dual-energy Xray absorptiometry, *FM* fat mass, *HV* high volume, *LV* low volume, *MJ-SJ* multi to single joint, *NP* non periodized, *RT* resistance training, *SF* skin folds, *SJ-MJ* single to multi joint*; < 5%* < 5% of truck fat gain

### Risk of bias assessment

Risk of bias assessment was completed for all included articles. The mean Downs and Black checklist score was 19.8 with the range between 14 and 27. Studies were then divided following different quality categories suggested by Tremblay et al. [56]. Six studies were placed into ‘high quality’ category, twelve studies were placed into ‘moderate quality’ category, six studies were placed into ‘lower quality’ category and two studies ware placed into poor quality category. The inter rater reliability coefficient between the assessors was 0.87, which as reported by Dawson and Trapp [57] corresponds to “very good agreement”. For detailed risk of bias reporting of each study, refer to Supplementary File. Level of evidence of the included studies ranged between 1B and 4, with 2 studies reaching level 1B, 6 studies reaching level 2B, 2 study reaching level 3B and 16 studies reaching level 4, suggesting an overall grade C of recommendation of the included manuscripts. A breakdown for each study is provided in Supplementary File.

### Meta-analytic synthesis of results

**Fig. 2:**
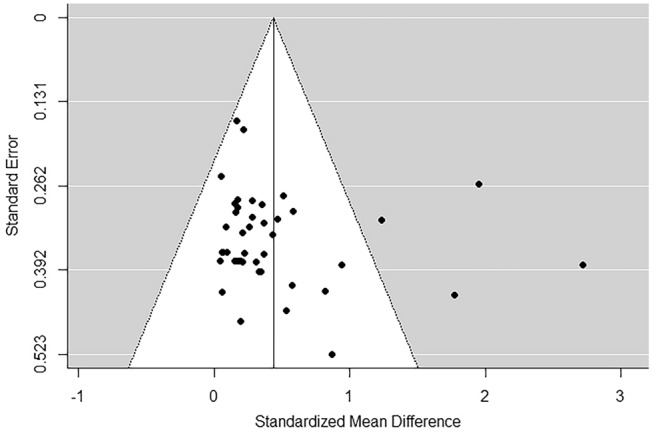
Funnel plot for publication bias evaluation for lean body mass

**Fig. 3:**
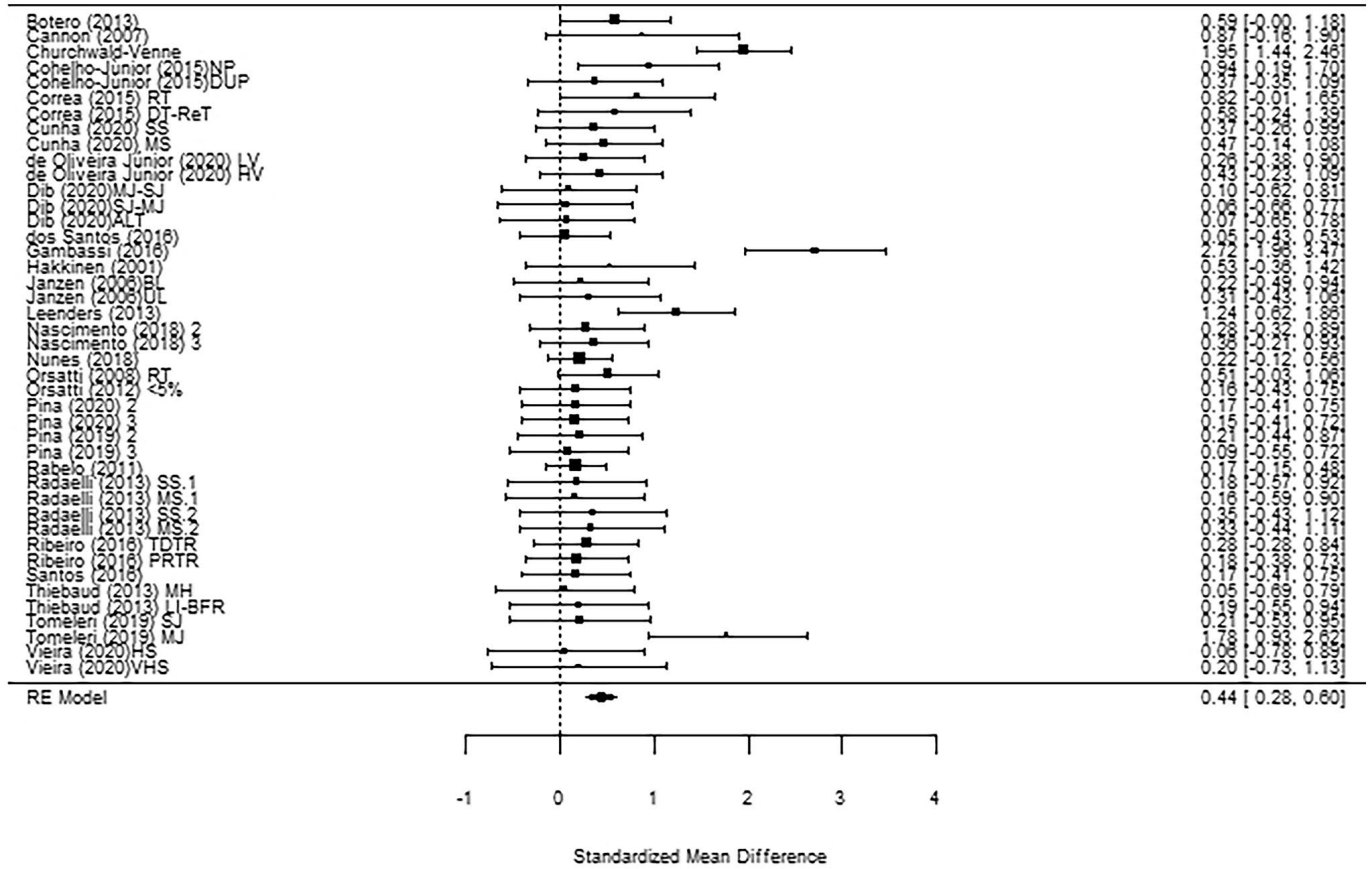
Figure shows the forest plot of the meta-analytic results regarding lean body mass. *2* Twice a week, *3* three times a week, *BL* bilateral, *DT-ReT* detraining-retraining, *DUP* ondulating periodized, *HS* High supervised, *HV* high volume, *LI-BFR* low load blood flow restriction exercise, *LV* low volume, *MH* moderate to high intensity elastic band resistance exercise, *MJ* multi joint, *MS* multiple-set resistance training, *MV* muscle volume, *NP* non periodized, *PR* pyramid, *RF* rectus femoris, *RT* resistance training, *SJ* single joint, *SS* single-set resistance training, *TD* traditional, *UL* unilateral, *VL* vastus lateralis, *VHS* very high supervised*; < 5%* < 5% of truck fat gain

**Fig. 4:**
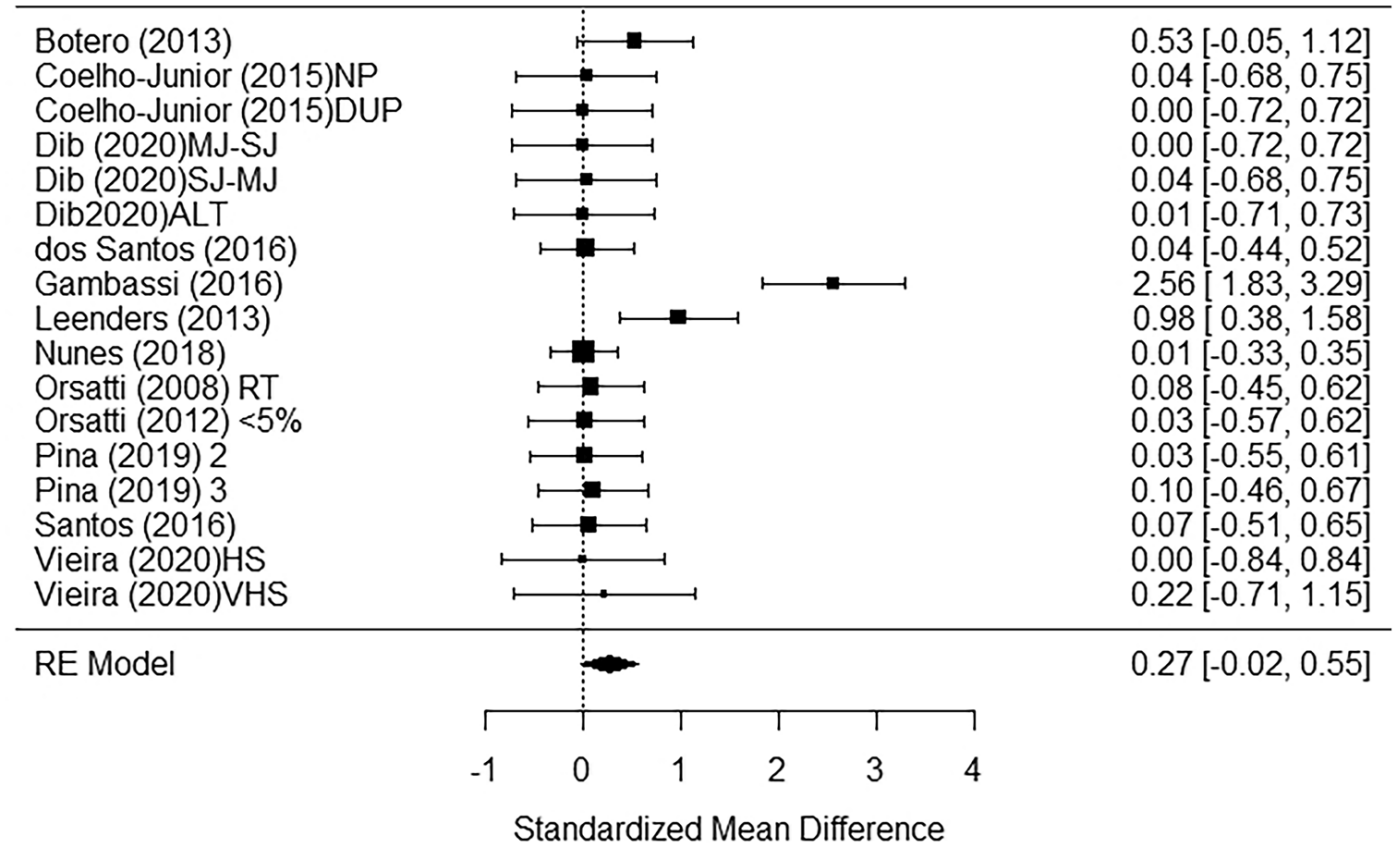
Figure shows the forest plot of the meta-analytic results regarding fat body mass. *2* Twice a week, *3* three times a week, *ALT* alternating upper and lower body, *DUP* periodized, *MJ-SJ* multi to single joint, *NP* non periodized, *RT* resistance training, *SJ-MJ* single to multi joint*; < 5%* < 5% of truck fat gain

The meta-analysis was performed on lean body mass and fat body mass. First of all, funnel plots did not show any publication bias (Fig. 2 shows lean body mass). Concerning lean body mass, a significant small-to-medium increase in the post-measurement was observed across *k* = 43 effects (SMD = 0.44; 95% CI 0.28; 0.60; *p* < 0.0001) (Fig. 3). Heterogeneity of the study resulted significant, with a Q(*df* = 42) = 109.95, *p* = 0.0001. Regarding fat body mass, no significant effect was detected across *k* = 17 effects (SMD = 0.27; 95% CI − 0.02; 0. 55; *p* = 0.07) (Fig. 4). Since the retrieved effects were homogeneous, we decided to calculate moderator analysis through mixed effects model. A moderator analysis was performed concerning age of the participants (defining two categories: below 65 and above 65 years of age), intervention length, number of weekly sessions and number of exercises proposed, highlighting no significant difference in the retrieved effects, neither for lean nor fat body mass.

The results of the sensitivity analyses showed that considering only results from DXA returned a SMD = 0.36 (95% CI 0.19–0.53, *p* < 0.0001), therefore very similar to our main results. The second sensitivity analysis was performed excluding the poor-quality studies, with a SMD = 0.45 (95% CI 0.27–0.63, *p* < 0.0001), indicating that poor-quality studies did not affect the average effect size.

## Discussion

This review article aimed to identify and analyze manuscripts regarding RT and hypertrophic responses in a postmenopausal and elderly adult female population. Our main findings highlight that all the analyzed RT protocols were able to moderately increase muscle mass in the sampled populations, despite differences in intervention length and assessment procedures. These effects however, are small-to-moderate (SMD = 0.44). Interestingly, no difference in lean body mass increase was present regarding age, weekly frequency and intervention length. Therefore, we could not identify a minimum dose–response for lean muscle mass improvement in the retrieved studies regarding RT. A systematic review and meta-analysis by Schoenfeld et al. [58] aimed to identify the main training parameters in order to increase strength and hypertrophic adaptations in a general population. The study has concluded that intensity is determinant in strength increases while volume can be modulated over different spectrums to promote muscle hypertrophy. These findings may provide explanation to our main results, since the included protocols had similar intensities and volume (being that the majority of studies were performing between 8 and 12 repetitions with an intensity of around 60% of 1RM). Another meta-analysis [59] which evaluated the training frequency of RT programs on gains in muscular strength has concluded that increased frequency is linked to increases in strength. However, when age groups were analyzed, only young adults seemed to benefit from increased RT frequency, while older adults did not. However, the results of this latter study only took into account measures of strength and not muscle mass. Nevertheless, evidence of a dose–response relationships in the elderly (taking into account both male and female) exists, suggesting that 2 sessions per week, performing 2 to 3 sets of 8 exercises, is effective in promoting strength and to modify muscle morphology [60]. The suggested protocol almost overlaps the mean reported data present in Table 1, which could explain the homogeneity of the results regarding lean body mass improvements observed across the retrieved studies.

It is important to note that increased muscle mass does not necessarily imply a causal relation with strength improvements [61] since the mechanisms responsible for strength development and muscle hypertrophy are different in nature [62, 63]. For example strength improvements as a result of increased neural drive are observed well before muscle hypertrophy as the result of increased motor unit firing rate or agonist–antagonist co-activation [62], while muscle hypertrophy is mainly stimulated by metabolic stress and mechanical tension which then activate intracellular pathways inducing muscle growth[63]. Although not a primary outcome of this review, as reported in Table 2, increases in strength were also observed for bench press, chest press, leg press and knee extension exercises.

The small effects highlighted by the meta-analytic synthesis, seem to be in line with the most recent scientific evidence, since as a consequence of aging, increased anabolic resistance, diminished muscle regeneration, impaired muscle activation and a reduction of the number of motor units are frequently observed [64]. However, precisely for these reasons it is important to engage postmenopausal and elderly women in RT programs, in order to improve muscle mass and strength, to reduce the risk of injury, and improve quality of life during the aging process [65].

Other analyzed aspect in this review was fat mass, which did not show any difference as a consequence of RT. Despite the general agreement regarding improved muscle mass to RT, there are still controversial reportings regarding fat mass [66], since some authors advocate decreases [67], while others do not [35] in this specific population.

It is important to outline that only the study by Nascimento et al. [34] considered dietary intake along with the RT protocol. Although nutritional aspects of training and recovery go outside the scope of this study, we cannot neglect the importance of proper diet in muscle hypertrophy [68], especially for post-menopausal women in which a correct nutritional regimen is recommended [21, 69], since the frequent dyslipidemic profiles observed [66]. In addition to nutritional status, other important consideration needs to be outlined regarding hormonal replacement therapy that may be prescribed as a form of prevention for estrogen deficiency. None of the included participants were undergoing hormonal therapy. To be noted that six studies [32, 37, 40, 50, 52, 53] did not specify if the participants were using hormonal replacement therapy. Therefore, the results of this study may primarily be attributable to the effects of the RT interventions.

Concerning clinical application, Nelson [70] and colleagues have shown that adults who do not take part in regular RT lose on average 0.46 kg of muscle per year from the age of 50. Additionally, sedentary subjects have reported a 50% reduction in fast-twitch muscle fibers by the age of 80 [27, 71]. These review findings are particularly important for post-menopausal and older women who are more susceptible to sarcopenia than men [72, 73]. Thus, creating exercise prescription that will induce hypertrophy, specifically in postmenopausal and older women could represent an efficient strategy to counteract the effects of sarcopenia and would contribute to overall better quality of life in adult women [74].

The strengths of this review embedded a comprehensive search strategy, stringent predetermined inclusion and exclusion criteria and thorough analyses of each article included. We included articles that examined direct and indirect measures of muscle hypertrophy and focused on incremental changes in the musculature trained in postmenopausal and older adult women. The results of our study provide evidence that RT may possibly counteract the effects of sarcopenia.

Nevertheless, our study is not without limitations, including the various types of outcome measurements sampled with heterogeneous methods. Despite significant efforts to identify appropriate techniques for muscle mass quantification [75, 76], a consensus regarding a gold standard procedure still needs to be defined. A recent review article has proposed dual energy X‐ray absorptiometry as a reference technique [77], taking into account advantages and disadvantages across available muscle mass measures. Therefore, data interpretation should always consider the principles behind each measurement technique before comparisons. One of the main limits, which neither dual energy X‐ray absorptiometry nor other adopted methods as CT scans, bioimpedance analysis and ultrasound evaluations, are able to detect fatty infiltrations within muscles, which is a common process caused by aging [77], which could lead to overestimation of muscle mass [78] in elderly or obese people.

Other aspects which needs to be considered are the relatively short intervention duration (mean 16 weeks) in the majority of studies, and study designs, since only three studies were randomized control trials while the majority were case series, therefore leading to a general low level of evidence. In addition, despite the single study results seem to be not homogeneous, no substantial differences in intervention length and assessment methods were present. Therefore, the moderator analysis performed could not identify a minimum dose–response to RT.

## Conclusion

Based on the data acquired through our systematic literature search, RT protocols are able to moderately increase muscle mass in post-menopausal and elderly woman but not to reduce fat mass. Exercise frequency, number of exercises per training session, protocol length and age of the participants do not seem to significantly moderate the evaluated effects. Research needs to concentrate on defining training parameters as volume and intensity in order to help sport professionals to more effectively program RT protocols which would in turn counteract the effects of sarcopenia.

## Supplementary Information

Below is the link to the electronic supplementary material.

Supplementary file1 (DOCX 51 KB)
