# Supplementary material for: The effect of resistance training programs on lean body mass in postmenopausal and elderly women: a meta-analysis of observational studies
Source: Aging Clin Exp Res. 2021 Apr 20;33(11):2941–52. doi: 10.1007/s40520-021-01853-8 (PMC8595144; doi:10.1007/s40520-021-01853-8)
Supplement: Supplementary file 1 — Supplementary file1 (DOCX 51 KB) [file 40520_2021_1853_MOESM1_ESM.docx]

**Appendix**

The Appendix contains

- a. Details of the literature search for one database.

- b. Downs and Black scores of each investigator.

- c. Downs and Black total scores for each study.

- d**.** Levels of evidence and grades of recommendations.

- e. Synthesis of excluded manuscripts.

| **a.** Details of the literature search for one database | | |
| --- | --- | --- |
| **Population** | | |
| Post-menopausal woman age ranging between 50 and 80 years of age [‘post-menopausal’, ‘elderly woman’, ‘elderly female’] | | |
| **Interventions** | | |
| Resistance training programs [‘resistance training’, ‘resistance exercise’, ‘strength training’] | | |
| **Comparators** | | |
| Other treatment, no treatment. | | |
| **Outcomes** | | |
| Variation in skeletal muscle mass measured with appropriate instruments [‘hypertrophy’, ‘muscle mass’, ‘lean tissue’] | | |
| **Study design** | | |
| Original articles | | |
| **Limitations** | | |
| English manuscripts, peer-review articles, published between January 2000 and November 2020, systematic reviews and meta-analysis. | | |
| **Keywords** | | |
| 1. Hypertrophy | | |
| 2. Muscle Mass | | |
| 3. Lean Tissue | | |
| 4. Resistance Training | | |
| 5. Strength Training | | |
| 6. Resistance Exercise | | |
| 7. Elderly woman | | |
| 8. Elderly Female | | |
| 9. Post-Menopausal | | |
| **Pubmed** | | |
| # | Searches | Results |
| 1 | 1 AND 4 AND 7 | 44 |
| 2 | 1 AND 4 AND 8 | 93 |
| 3 | 1 AND 4 AND 9 | 0 |
| 4 | 1 AND 5 AND 7 | 55 |
| 5 | 1 AND 5 AND 8 | 110 |
| 6 | 1 AND 5 AND 9 | 0 |
| 7 | 1 AND 6 AND 7 | 47 |
| 8 | 1 AND 6 AND 8 | 96 |
| 9 | 1 AND 6 AND 9 | 0 |
| 10 | 2 AND 4 AND 7 | 254 |
| 11 | 2 AND 4 AND 8 | 513 |
| 12 | 2 AND 4 AND 9 | 4 |
| 13 | 2 AND 5 AND 7 | 351 |
| 14 | 2 AND 5 AND 8 | 702 |
| 15 | 2 AND 5 AND 9 | 6 |
| 16 | 2 AND 6 AND 7 | 278 |
| 17 | 2 AND 6 AND 8 | 571 |
| 18 | 2 AND 6 AND 9 | 4 |
| 19 | 3 AND 4 AND 7 | 81 |
| 20 | 3 AND 4 AND 8 | 144 |
| 21 | 3 AND 4 AND 9 | 1 |
| 22 | 3 AND 5 AND 7 | 97 |
| 23 | 3 AND 5 AND 8 | 177 |
| 24 | 3 AND 5 AND 9 | 2 |
| 25 | 3 AND 6 AND 7 | 88 |
| 26 | 3 AND 6 AND 8 | 161 |
| 27 | 3 AND 6 AND 9 | 1 |
| The search was conducted up to the 11^th^ of November 2020, the same approach was adopted for all the screened database. | | |

| **b.** Downs and Black scores of each investigator. | | | | | | | | | | | | | |
| --- | --- | --- | --- | --- | --- | --- | --- | --- | --- | --- | --- | --- | --- |
| **Author** | **year** | **R (_/11)** | | **EV (_/3)** | | **IVB (_/7)** | | **IVC (_/6)** | | **P (_/5)** | | **T (_/32)** | |
| **Original study** |  | I1 | I2 | I1 | I2 | I1 | I2 | I1 | I2 | I1 | I2 | I1 | I2 |
| *Botero et al* | 2013 | 8 | 10 | 1 | 1 | 4 | 2 | 1 | 1 | 0 | 0 | 14 | 14 |
| *Cannon et al* | 2007 | 6 | 6 | 0 | 1 | 3 | 3 | 2 | 0 | 3 | 5 | 14 | 15 |
| *Churchward-Venne et al* | 2015 | 8 | 9 | 1 | 2 | 3 | 4 | 3 | 3 | 3 | 3 | 18 | 21 |
| *Coelho-Júnior et al* | 2015 | 10 | 9 | 2 | 1 | 5 | 6 | 5 | 5 | 4 | 3 | 26 | 24 |
|  |  |  |  |  |  |  |  |  |  |  |  |  |  |
| *Correa et al* | 2015 | 6 | 8 | 0 | 2 | 4 | 5 | 1 | 2 | 3 | 3 | 14 | 20 |
| *Cunha et al* | 2018 | 9 | 6 | 2 | 2 | 6 | 3 | 5 | 3 | 4 | 4 | 26 | 18 |
| *de Oliveira Júnior et al* | 2020 | 11 | 9 | 1 | 2 | 5 | 5 | 4 | 6 | 5 | 5 | 26 | 27 |
| *Dib et al* | 2020 | 10 | 11 | 2 | 2 | 4 | 4 | 4 | 6 | 3 | 3 | 23 | 26 |
| *dos Santos et al* | 2016 | 8 | 9 | 1 | 1 | 4 | 3 | 2 | 1 | 3 | 0 | 18 | 14 |
| *Gambassi et al* | 2016 | 8 | 7 | 1 | 1 | 3 | 4 | 2 | 4 | 2 | 2 | 16 | 18 |
| *Hakkinen et al* | 2001 | 6 | 6 | 0 | 2 | 4 | 3 | 0 | 1 | 5 | 5 | 15 | 17 |
| *Jenzen et al* | 2006 | 10 | 8 | 2 | 1 | 4 | 4 | 5 | 5 | 3 | 3 | 24 | 21 |
| *Leenders et al* | 2013 | 10 | 8 | 0 | 1 | 4 | 4 | 3 | 2 | 4 | 4 | 21 | 18 |
| *Nascimento et al* | 2018 | 10 | 7 | 2 | 2 | 5 | 5 | 4 | 3 | 4 | 4 | 25 | 21 |
| *Nunes et al* | 2018 | 8 | 10 | 1 | 1 | 4 | 4 | 3 | 3 | 4 | 4 | 20 | 22 |
| *Orsatti et al* | 2008 | 9 | 6 | 0 | 0 | 4 | 3 | 4 | 2 | 4 | 4 | 21 | 15 |
| *Orsatti et al* | 2012 | 9 | 7 | 0 | 2 | 4 | 3 | 2 | 2 | 4 | 4 | 19 | 18 |
| *Pina et al* | 2020 | 8 | 11 | 1 | 2 | 4 | 3 | 4 | 5 | 3 | 5 | 20 | 26 |
| *Pina et al* | 2019 | 7 | 9 | 2 | 2 | 3 | 4 | 3 | 4 | 5 | 4 | 20 | 23 |
| *Rabelo et al* | 2011 | 7 | 10 | 1 | 1 | 4 | 4 | 3 | 2 | 4 | 4 | 19 | 21 |
| *Radaelli et al* | 2015 | 6 | 6 | 0 | 0 | 3 | 3 | 2 | 2 | 3 | 3 | 14 | 14 |
| *Ribeiro et al* | 2015 | 7 | 6 | 2 | 2 | 5 | 5 | 3 | 3 | 4 | 4 | 21 | 20 |
| *Santos et al* | 2017 | 10 | 8 | 2 | 2 | 5 | 4 | 1 | 5 | 5 | 4 | 23 | 23 |
|  |  |  |  |  |  |  |  |  |  |  |  |  |  |
| *Thiebaud et al* | 2017 | 9 | 7 | 0 | 3 | 4 | 5 | 1 | 3 | 2 | 4 | 16 | 22 |
| *Tomeleri et al* | 2019 | 9 | 9 | 1 | 0 | 5 | 4 | 4 | 4 | 4 | 3 | 23 | 20 |
| *Vieira et al* | 2020 | 7 | 7 | 1 | 0 | 4 | 3 | 3 | 4 | 4 | 4 | 19 | 18 |
| Mean |  | 8.2 | | 1.19 | | 3.98 | | 2.98 | | 3.52 | | 19.8 | |
| R: Reporting; EV: External Validity; IVC: Internal validity confounding; IVB: Internal validity bias; P: Power; T: Total score; I1: Investigator 1; I2: Investigator 2. | | | | | | | | | | | | | |

| \| **c.** Downs and Black total scores for each study. \| \| \| \| \| \| --- \| --- \| --- \| --- \| --- \| \| Study \| High quality (23-32) \| Moderate quality (19-22) \| Lower quality (15-18) \| Poor quality (≤14) \| \| Botero et al \| / \| / \| / \| 14 \| \| Cannon et al \| / \| / \| 14.5 \| / \| \| Churchward-Venne et al \| / \| 19.5 \| / \| / \| \| Coelho-Júnior et al \| 25 \| / \| / \| / \| \|  \|  \|  \|  \|  \| \| Correa et al \| / \| / \| 16 \| / \| \| Cunha et al \| / \| 22 \| / \| / \| \| de Oliveira Júnior et al \| 26.5 \| / \| / \| / \| \| Dib et al \| 24.5 \| / \| / \| / \| \| dos Santos et al \| / \| / \| 16 \| / \| \| Gambassi et al \| / \| / \| 17 \| / \| \| Hakkinen et al \| / \| / \| 16 \| / \| \| Jenzen et al \| / \| 22.5 \| / \| / \| \| Leenders et al \| / \| 19.5 \| / \| / \| \| Nascimento et al \| 23 \| / \| / \| / \| \| Nunes et al \| / \| 21 \| / \| / \| \| Orsatti et al \| / \| / \| 18 \| / \| \| Orsatti et al \| / \| 18.5 \| / \| / \| \| Pina et al \| 23 \| / \| / \| / \| \| Pina et al \| / \| 21.5 \| / \| / \| \| Rabelo et al \| / \| 20 \| / \| / \| \| Radaelli et al \| / \| / \| / \| 14 \| \| Ribeiro et al \| / \| 20.5 \| / \| / \| \| Santos et al \| 23 \| / \| / \| / \| \|  \|  \|  \|  \|  \| \| Thiebaud et al \| / \| 19 \| / \| / \| \| Tomeleri et al \| / \| 21.5 \| / \| / \| \| Vieira et al \| / \| 18.5 \| / \| / \| |
| --- | --- | --- | --- | --- | --- | --- | --- | --- | --- | --- | --- | --- | --- | --- | --- | --- | --- | --- | --- | --- | --- | --- | --- | --- | --- | --- | --- | --- | --- | --- | --- | --- | --- | --- | --- | --- | --- | --- | --- | --- | --- | --- | --- | --- | --- | --- | --- | --- | --- | --- | --- | --- | --- | --- | --- | --- | --- | --- | --- | --- | --- | --- | --- | --- | --- | --- | --- | --- | --- | --- | --- | --- | --- | --- | --- | --- | --- | --- | --- | --- | --- | --- | --- | --- | --- | --- | --- | --- | --- | --- | --- | --- | --- | --- | --- | --- | --- | --- | --- | --- | --- | --- | --- | --- | --- | --- | --- | --- | --- | --- | --- | --- | --- | --- | --- | --- | --- | --- | --- | --- | --- | --- | --- | --- | --- | --- | --- | --- | --- | --- | --- | --- | --- | --- | --- | --- | --- | --- | --- | --- | --- | --- | --- | --- | --- | --- | --- | --- | --- | --- |

| **d.** Levels of evidence and grades of recommendations | | |
| --- | --- | --- |
| **Author** | Level of Evidence | Grades of Recommendation |
| Botero et al | 4 | C |
| Cannon et al | 4 | C |
| Churchward-Venne et al | 4 | C |
| Coelho-Júnior et al | 1B | A |
|  |  |  |
| Correa et al | 4 | C |
| Cunha et al | 1B | A |
| de Oliveira Júnior et al | 2B | B |
| Dib et al | 2B | B |
| dos Santos et al | 4 | C |
| Gambassi et al | 3B | B |
| Hakkinen et al | 4 | C |
| Jenzen et al | 2B | B |
| Leenders et al | 4 | C |
| Nascimento et al | 2B | B |
| Nunes et al | 4 | C |
| Orsatti et al | 2B | B |
| Orsatti et al | 4 | C |
| Pina et al | 4 | C |
| Pina et al | 4 | C |
| Rabelo et al | 3B | B |
| Radaelli et al | 4 | C |
| Ribeiro et al | 4 | C |
| Santos et al | 4 | C |
|  |  |  |
| Thiebaud et al | 4 | C |
| Tomeleri et al | 1B | A |
| Vieira et al | 4 | C |
| Level of Evidence 1A= Systematic review (with homogeneity) of RCTs; 1B=Individual RCT (with narrow confidence intervals); 1C=All or none study; 2A=Systematic review (with homogeneity) of cohort studies; 2B= Individual Cohort study (including low quality RCT, e.g. <80% follow-up); 2C=“Outcomes” research; Ecological studies; 3A= Systematic review (with homogeneity) of case-control studies; 3B=Individual Case-control study; 4= Case series (and poor quality cohort and case-control study; 5=Expert opinion without explicit critical appraisal or based on physiology bench research or “first principles” *From the Centre for Evidence-Based Medicine, <http://www.cebm.net>. Grades of Recommendation A= Level 1, Strong recommendation; B=Levels 2, 3 or 4, Recommendation; C=Levels 2, 3 or 4, Option; D=Level 5, Option. From American Society of Plastic Surgeons Evidence-based clinical practice guidelines. Available at: <https://www.plasticsurgery.org/documents/medical-professionals/health-policy/evidence-practice/ASPS-Scale-for-Grading-Recommendations.pdf>. Accessed February 12, 2021. | | |

| e. Synthesis of excluded manuscripts | | |
| --- | --- | --- |
| # | Article | Reason |
| 1 | Avila JJ, Gutierres JA, Sheehy ME, Lofgren IE, Delmonico MJ. Effect of moderate intensity resistance training during weight loss on body composition and physical performance in overweight older adults. Eur J Appl Physiol. 2010 Jun;109(3):517-25. doi: 10.1007/s00421-010-1387-9. Epub 2010 Feb 19. PMID: 20169360. | Not RT protocols or women |
| 2 | Fragala MS, Fukuda DH, Stout JR, Townsend JR, Emerson NS, Boone CH, Beyer KS, Oliveira LP, Hoffman JR. Muscle quality index improves with resistance exercise training in older adults. Exp Gerontol. 2014 May;53:1-6. doi: 10.1016/j.exger.2014.01.027. Epub 2014 Feb 6. PMID: 24508922. | Not RT protocols or women |
| 3 | Gray M, Powers M, Boyd L, Garver K. Longitudinal comparison of low- and high-velocity resistance training in relation to body composition and functional fitness of older adults. Aging Clin Exp Res. 2018 Dec;30(12):1465-1473. doi: 10.1007/s40520-018-0929-6. Epub 2018 Mar 22. PMID: 29569116. | Not RT protocols or women |
| 4 | Huang SW, Ku JW, Lin LF, Liao CD, Chou LC, Liou TH. Body composition influenced by progressive elastic band resistance exercise of sarcopenic obesity elderly women: a pilot randomized controlled trial. Eur J Phys Rehabil Med. 2017 Aug;53(4):556-563. doi: 10.23736/S1973-9087.17.04443-4. Epub 2017 Jan 12. PMID: 28084062. | Not RT protocols or women |
| 5 | Kemmler W, von Stengel S, Engelke K, Häberle L, Mayhew JL, Kalender WA. Exercise, body composition, and functional ability: a randomized controlled trial. Am J Prev Med. 2010 Mar;38(3):279-87. doi: 10.1016/j.amepre.2009.10.042. PMID: 20171529. | Not RT protocols or women |
| 6 | Lambert BS, Shimkus KL, Fluckey JD, Riechman SE, Greene NP, Cardin JM, Crouse SF. Anabolic responses to acute and chronic resistance exercise are enhanced when combined with aquatic treadmill exercise. Am J Physiol Endocrinol Metab. 2015 Feb 1;308(3):E192-200. doi: 10.1152/ajpendo.00689.2013. Epub 2014 Nov 25. PMID: 25425002. | Not RT protocols or women |
| 7 | Lee JS, Kim CG, Seo TB, Kim HG, Yoon SJ. Effects of 8-week combined training on body composition, isokinetic strength, and cardiovascular disease risk factors in older women. Aging Clin Exp Res. 2015 Apr;27(2):179-86. doi: 10.1007/s40520-014-0257-4. Epub 2014 Jul 6. PMID: 24997614. | Not RT protocols or women |
| 8 | Lindemann U, Mohr C, Machann J, Blatzonis K, Rapp K, Becker C Association between Thigh Muscle Volume and Leg Muscle Power in Older Women. 2016 PLoS ONE 11(6): e0157885. https://doi.org/10.1371/journal.pone.0157885 | Not RT protocols or women |
| 9 | Lixandrão ME, Damas F, Chacon-Mikahil MP, Cavaglieri CR, Ugrinowitsch C, Bottaro M, Vechin FC, Conceição MS, Berton R, Libardi CA. Time Course of Resistance Training-Induced Muscle Hypertrophy in the Elderly. J Strength Cond Res. 2016 Jan;30(1):159-63. doi: 10.1519/JSC.0000000000001019. PMID: 26110345. | Not RT protocols or women |
| 10 | Oh SL, Kim HJ, Woo S, Cho BL, Song M, Park YH, Lim JY, Song W. Effects of an integrated health education and elastic band resistance training program on physical function and muscle strength in community-dwelling elderly women: Healthy Aging and Happy Aging II study. Geriatr Gerontol Int. 2017 May;17(5):825-833. doi: 10.1111/ggi.12795. Epub 2016 May 3. PMID: 27138245. | Not RT protocols or women |
| 11 | Piastra G, Perasso L, Lucarini S, Monacelli F, Bisio A, Ferrando V, Gallamini M, Faelli E, Ruggeri P. Effects of Two Types of 9-Month Adapted Physical Activity Program on Muscle Mass, Muscle Strength, and Balance in Moderate Sarcopenic Older Women. Biomed Res Int. 2018 Oct 18;2018:5095673. doi: 10.1155/2018/5095673. PMID: 30420965; PMCID: PMC6211206. | Not RT protocols or women |
| 12 | Ramírez Villada, Jhon Fredy, León Ariza, Henry Humberto, Jiménez, Alveiro Sánchez and Sepúlveda, Celia Mónica. "Alterations in body composition, capillary glucose and functionality during explosive strength training in older women" International Journal on Disability and Human Development, vol. 15, no. 3, 2016, pp. 251-259 | Not RT protocols or women |
| 13 | Robinson MM, Dasari S, Konopka AR, Johnson ML, Manjunatha S, Esponda RR, Carter RE, Lanza IR, Nair KS. Enhanced Protein Translation Underlies Improved Metabolic and Physical Adaptations to Different Exercise Training Modes in Young and Old Humans. Cell Metab. 2017 Mar 7;25(3):581-592. doi: 10.1016/j.cmet.2017.02.009. PMID: 28273480; PMCID: PMC5423095. | Not RT protocols or women |
| 14 | Romero-Arenas S, Blazevich AJ, Martínez-Pascual M, Pérez-Gómez J, Luque AJ, López-Román FJ, Alcaraz PE. Effects of high-resistance circuit training in an elderly population. Exp Gerontol. 2013 Mar;48(3):334-40. doi: 10.1016/j.exger.2013.01.007. Epub 2013 Jan 23. PMID: 23352954. | Not RT protocols or women |
| 15 | Sims ST, Kubo J, Desai M, et al. Changes in physical activity and body composition in postmenopausal women over time. Med Sci Sports Exerc. 2013;45(8):1486-1492. doi:10.1249/MSS.0b013e31828af8bd | Not RT protocols or women |
| 16 | Socha M, Frączak P, Jonak W, Sobiech KA. Effect of resistance training with elements of stretching on body composition and quality of life in postmenopausal women. Prz Menopauzalny. 2016;15(1):26-31. doi:10.5114/pm.2016.58770 | Not RT protocols or women |
| 17 | Timmons JF, Minnock D, Hone M, Cogan KE, Murphy JC, Egan B. Comparison of time-matched aerobic, resistance, or concurrent exercise training in older adults. Scand J Med Sci Sports. 2018 Nov;28(11):2272-2283. doi: 10.1111/sms.13254. Epub 2018 Jul 16. PMID: 29947107. | Not RT protocols or women |
| 18 | Tsuzuku S, Kajioka T, Sakakibara H, Shimaoka K. Slow movement resistance training using body weight improves muscle mass in the elderly: A randomized controlled trial. Scand J Med Sci Sports. 2018 Apr;28(4):1339-1344. doi: 10.1111/sms.13039. Epub 2018 Jan 30. PMID: 29247985. | Not RT protocols or women |
| 19 | Turpela M, Häkkinen K, Haff GG, Walker S. Effects of different strength training frequencies on maximum strength, body composition and functional capacity in healthy older individuals. Exp Gerontol. 2017 Nov;98:13-21. doi: 10.1016/j.exger.2017.08.013. Epub 2017 Aug 15. PMID: 28821427. | Not RT protocols or women |
| 20 | Van Roie E, Delecluse C, Coudyzer W, Boonen S, Bautmans I. Strength training at high versus low external resistance in older adults: effects on muscle volume, muscle strength, and force-velocity characteristics. Exp Gerontol. 2013 Nov;48(11):1351-61. doi: 10.1016/j.exger.2013.08.010. Epub 2013 Aug 30. PMID: 23999311. | Not RT protocols or women |
| 21 | Vechin FC, Libardi CA, Conceição MS, Damas FR, Lixandrão ME, Berton RP, Tricoli VA, Roschel HA, Cavaglieri CR, Chacon-Mikahil MP, Ugrinowitsch C. Comparisons between low-intensity resistance training with blood flow restriction and high-intensity resistance training on quadriceps muscle mass and strength in elderly. J Strength Cond Res. 2015 Apr;29(4):1071-6. doi: 10.1519/JSC.0000000000000703. PMID: 25264670. | Not RT protocols or women |
| 22 | von Stengel S, Kemmler W, Engelke K, Kalender WA. Effect of whole-body vibration on neuromuscular performance and body composition for females 65 years and older: a randomized-controlled trial. Scand J Med Sci Sports. 2012 Feb;22(1):119-27. doi: 10.1111/j.1600-0838.2010.01126.x. Epub 2010 May 24. PMID: 20500555. | Not RT protocols or women |
| 23 | Watanabe Y, Tanimoto M, Oba N, Sanada K, Miyachi M, Ishii N. Effect of resistance training using bodyweight in the elderly: Comparison of resistance exercise movement between slow and normal speed movement. Geriatr Gerontol Int. 2015 Dec;15(12):1270-7. doi: 10.1111/ggi.12427. Epub 2015 Jan 17. PMID: 25598234. | Not RT protocols or women |
| 24 | Ahtiainen JP, Walker S, Peltonen H, et al. Heterogeneity in resistance training-induced muscle strength and mass responses in men and women of different ages. Age (Dordr). 2016;38(1):10. doi:10.1007/s11357-015-9870-1 | Missing Data |
| 25 | Carneiro, M.A.S., de Oliveira Júnior, G.N., de Sousa, J.F.R. et al. Effects of cluster training sets on muscle power and force–velocity relationship in postmenopausal women. Sport Sci Health 16, 257–265 (2020). https://doi.org/10.1007/s11332-019-00599-1 | Missing Data |
| 26 | Kosek DJ, Kim JS, Petrella JK, Cross JM, Bamman MM. Efficacy of 3 days/wk resistance training on myofiber hypertrophy and myogenic mechanisms in young vs. older adults. J Appl Physiol (1985). 2006 Aug;101(2):531-44. doi: 10.1152/japplphysiol.01474.2005. Epub 2006 Apr 13. PMID: 16614355. | Missing Data |
| 27 | Strandberg E, Edholm P, Ponsot E, Wåhlin-Larsson B, Hellmén E, Nilsson A, Engfeldt P, Cederholm T, Risérus U, Kadi F. Influence of combined resistance training and healthy diet on muscle mass in healthy elderly women: a randomized controlled trial. J Appl Physiol (1985). 2015 Oct 15;119(8):918-25. doi: 10.1152/japplphysiol.00066.2015. Epub 2015 Sep 3. PMID: 26338453. | Missing Data |
| 28 | Urzi F, Marusic U, Ličen S, Buzan E. Effects of Elastic Resistance Training on Functional Performance and Myokines in Older Women-A Randomized Controlled Trial. J Am Med Dir Assoc. 2019 Jul;20(7):830-834.e2. doi: 10.1016/j.jamda.2019.01.151. Epub 2019 Mar 20. PMID: 30902674. | Missing Data |
| 29 | Vikberg S, Sörlén N, Brandén L, Johansson J, Nordström A, Hult A, Nordström P. Effects of Resistance Training on Functional Strength and Muscle Mass in 70-Year-Old Individuals With Pre-sarcopenia: A Randomized Controlled Trial. J Am Med Dir Assoc. 2019 Jan;20(1):28-34. doi: 10.1016/j.jamda.2018.09.011. Epub 2018 Nov 7. PMID: 30414822. | Missing Data |
| 30 | Kraemer WJ, Nindl BC, Ratamess NA, Gotshalk LA, Volek JS, Fleck SJ, Newton RU, and Hakkinen K. Changes in Muscle Hypertrophy in Women with Periodized Resistance Training. Medicine & Science in Sports & Exercise 36: 697-708, 2004. | Age of Participants |
| 31 | Mueller M, Breil FA, Vogt M, Steiner R, Lippuner K, Popp A, Klossner S, Hoppeler H, Däpp C. Different response to eccentric and concentric training in older men and women. Eur J Appl Physiol. 2009 Sep;107(2):145-53. doi: 10.1007/s00421-009-1108-4. Epub 2009 Jun 20. PMID: 19543908. | Age of participants |
| 32 | Pelzer T, Ullrich B, Pfeiffer M. Periodization effects during short-term resistance training with equated exercise variables in females. Eur J Appl Physiol. 2017 Mar;117(3):441-454. doi: 10.1007/s00421-017-3544-x. Epub 2017 Feb 3. PMID: 28160082. | Age of participants |
| 33 | Seynnes OR, de Boer M, Narici MV. Early skeletal muscle hypertrophy and architectural changes in response to high-intensity resistance training. J Appl Physiol (1985). 2007 Jan;102(1):368-73. doi: 10.1152/japplphysiol.00789.2006. Epub 2006 Oct 19. PMID: 17053104. | Age of participants |
| 34 | Zech A, Drey M, Freiberger E, Hentschke C, Bauer JM, Sieber CC, Pfeifer K. Residual effects of muscle strength and muscle power training and detraining on physical function in community-dwelling prefrail older adults: a randomized controlled trial. BMC Geriatr. 2012 Nov 7;12:68. doi: | Age of participants |
| 35 | B.S. Shaw, M. Gouveia, S. McIntyre, et al., Anthropometric and cardiovascular responses to hypertrophic resistance training in postmenopausal women, Menopause 23(11) (2016) 1176-1181. | Measurement technique |
| 36 | Delshad M, Ghanbarian A, Mehrabi Y, Sarvghadi F, Ebrahim K. Effect of Strength Training and Short-term Detraining on Muscle Mass in Women Aged Over 50 Years Old. Int J Prev Med. 2013;4(12):1386-1394. | Measurement technique |
| 37 | M.S. Conceição, V. Bonganha, F.C. Vechin, et al., Sixteen weeks of resistance training can decrease the risk of metabolic syndrome in healthy postmenopausal women, Clin Interv Aging 8 (2013) 1221-8. | Measurement technique |
| 38 | Pinto RS, Correa CS, Radaelli R, Cadore EL, Brown LE, Bottaro M. Short-term strength training improves muscle quality and functional capacity of elderly women. Age (Dordr). 2014 Feb;36(1):365-72. doi: 10.1007/s11357-013-9567-2. Epub 2013 Jul 24. PMID: 23881608; PMCID: PMC3889909. | Measurement technique |
| 39 | Liao CD, Tsauo JY, Lin LF, Huang SW, Ku JW, Chou LC, Liou TH. Effects of elastic resistance exercise on body composition and physical capacity in older women with sarcopenic obesity: A CONSORT-compliant prospective randomized controlled trial. Medicine (Baltimore). 2017 Jun;96(23):e7115. doi: 10.1097/MD.0000000000007115. PMID: 28591061; PMCID: PMC5466239. | Not physiological |
| 40 | Liao CD, Tsauo JY, Huang SW, Ku JW, Hsiao DJ, Liou TH. Effects of elastic band exercise on lean mass and physical capacity in older women with sarcopenic obesity: A randomized controlled trial. Sci Rep. 2018 Feb 2;8(1):2317. doi: 10.1038/s41598-018-20677-7. PMID: 29396436; PMCID: PMC5797161. | Not physiological |
| 41 | Dao E, Davis JC, Sharma D, Chan A, Nagamatsu LS, Liu-Ambrose T. Change in body fat mass is independently associated with executive functions in older women: a secondary analysis of a 12-month randomized controlled trial. PLoS One. 2013;8(1):e52831. doi:10.1371/journal.pone.0052831 | Unclear Protocol |
